# Survey of echinococcoses in southeastern Qinghai Province, China, and serodiagnostic insights of recombinant *Echinococcus granulosus* antigen B isoforms

**DOI:** 10.1186/s13071-019-3569-6

**Published:** 2019-06-26

**Authors:** Xiumin Han, Jeong-Geun Kim, Hu Wang, Huixia Cai, Xiao Ma, Duc Hieu Duong, Chun-Seob Ahn, Insug Kang, Yoon Kong

**Affiliations:** 1 Qinghai Provincial People’s Hospital, Xining, 810007 China; 2 Qinghai Province Institute for Endemic Diseases Prevention and Control, Qinghai Centers for Disease Prevention and Control, Xining, 811602 China; 3 Department of Molecular Parasitology, Samsung Medical Center, Sungkyunkwan University School of Medicine, Suwon, 16419 Korea; 4 Endemic Disease Administration Office, Qinghai Province Health and Family Planning Commission, Xining, 811602 China; 5 Department of Molecular Biology and Biochemistry, Kyung Hee University College of Medicine, Seoul, 02447 Korea

**Keywords:** Echinococcoses, *Echinococcus granulosus*, *E. multilocularis*, Qinghai-Tibetan Plateau, Ultrasonography, Antigen B isoforms, Immunoreactivity

## Abstract

**Background:**

Echinococcoses, caused by metacestodes of *Echinococcus granulosus* (cystic echinococcosis; CE) and *E. multilocularis* (alveolar echinococcosis; AE), represent major emerging parasitic diseases. These enzootic helminthiases invoke significant public health concerns and social burdens in endemic areas. The diseases are prevalent in the Qinghai-Tibetan Plateau, China, while community-based epidemiological studies have been scarcely reported. We surveyed echinococcosis patients in the southeastern Qinghai Province, China, to better understand the concurrent epidemiological situation in this area.

**Methods:**

During July and August of 2013 and 2014, we screened echinococcosis patients at Yushu and Golog Prefectures, Qinghai Province, China, in a diagnostic campaign. A total of 2856 people (male:female ratio, 1:1.12; mean age, 34.6 years; age range, 6–88 years) were ultrasonographically examined for the presence of hepatic echinococcal cysts. We also collected serum samples from patients and analyzed antibody reactivity against recombinant forms of diverse *E. granulosus* antigen Bs (rEgAgB1-5) by enzyme-linked immunosorbent assay.

**Results:**

We detected 134 patients whose imaging scans were compatible with CE (115 cases) and AE (20 patients). One patient might have been infected with both CE and AE. The overall incidence was 4.7% (CE, 4.0%; AE, 0.7%). A large proportion (67.5%) of CE patients was diagnosed at active and transitional CE1-CE3 stages in their late 30s. The AE cases were generally detected at advanced stage in patients at early 20s (60%). Analysis of the receiver operating characteristic curve and Youden’s index indicated that rEgAgB2 was the most promising biomarker, followed by rEgAgB3 and rEgAgB1. Overall, sensitivity and specificity of rEgAgB1-3 were 84.5–92.7% and 91.9–94.6%, respectively. rEgAgB4 and 5 showed low sensitivity with high cross-reactivity.

**Conclusions:**

Our results strongly suggest that disability-adjusted life years related to echinococcoses in Qinghai-Tibetan areas might be more serious than previously considered. Control and prevention strategy against CE and AE are highly required in these areas. In addition to ultrasonography, serological tests might provide supportive data. However, serological data should be carefully interpreted for differential diagnosis, especially in areas where both CE and AE are co-endemic.

**Electronic supplementary material:**

The online version of this article (10.1186/s13071-019-3569-6) contains supplementary material, which is available to authorized users.

## Background

A number of *Echinococcus* spp. cause echinococcoses during their metacestode stage, among which *Echinococcus granulosus* and *E. multilocularis* invoke major human diseases, cystic echinococcosis (CE) and alveolar echinococcosis (AE). Humans serve as an accidental intermediate host and are infected with these parasites by contracting eggs expelled from canine definitive hosts. Ingested eggs are activated in the small intestine, released into the bloodstream, and primarily end up lodged in the liver. *Echinococcus granulosus* metacestode typically forms a unilocular cyst that induces a space-occupying lesion whereas *E. multilocularis* metacestode progresses to a multivesiculated cystic mass that infiltrates into the nearby hepatic parenchyme with behavior resembling invasive malignant tumors. AE sometimes establishes metastatic lesion(s) in other remote tissues/organs [1].

Echinococcoses represent major emerging enzootic parasitic diseases, which have resulted in significant medical problems and public health concerns in endemic areas. CE is prevalent in nomadic areas of Europe, Central and Middle Asia, Africa, Australia, South America and northwestern China in association with dog-rearing environments [1]. Approximately four million people are infected and another 40 million people are at risk annually [2, 3]. AE is endemic to several communities in Eurasia, North America and northwestern China. The overall annual incidence of AE has been reported to be 0.2–6.0 per 100,000 inhabitants [4, 5]. AE exemplifies one of the most fatal helminthic diseases. Its lethality has been reported to be substantially increased if not treated properly [6]. In recent years, patients infected with *E. granulosus* and *E. multilocularis* have been increasingly detected in northern parts of China, including Xinjiang, Qinghai-Tibetan Plateau and Heilongjiang areas. In these areas, the seroprevalence rate of CE is approximately 5–30% of the inhabitants and that of AE is as high as 6% in some village populations [7, 8].

The diagnosis of echinococcoses largely depends on imaging scans and serological tests. Demonstration of typical lesions by ultrasonographic (US) examination has been the diagnosis of choice. US diagnosis has widely been used in both field survey and bedside diagnosis. The procedure is beneficial because it is easy to apply and is not invasive. Moreover, it has the definitive merit of providing an immediate diagnosis [9–12]. However, differential diagnosis of echinococcoses from hepatic cyst(s)/mass(es) with other etiologies such as hemorrhagic cyst, pyogenic/infectious abscess, cystadenoma, liver cirrhosis, primary hepatocellular carcinoma and metastatic tumors is often required and sometimes difficult, even for experienced physicians [13].

Serological tests might compensate for this shortcoming and provide additional evidence for echinococcoses [14–16]. Several antigenic proteins have been characterized to analyze their serodiagnostic performance, including *E. granulosus* antigen 5 (EgAg5), diverse antigen B subunits (EgAgBs) and endophilin B1. These molecules show sensitive antibody responses against patient sera from CE cases. However, the diagnostic applicability of EgAg5 and endophilin B1 is compromised by the lack of specificity [17, 18]. EgAgB proteins are abundantly found in hydatid fluid (HF) and are shown to induce strong antibody responses to sera from infected individuals [14–16]. *Echinococcus multilocularis* metacestodes develop into multivesiculated masses containing minute vesicular fluid. However, *E. multilocularis* antigen B proteins (EmAgBs), which are orthologs of EgAgBs, are also found in *E. multilocularis* HF [19]. EmAgB3 is abundantly expressed in the early developmental stage and exhibits specific antibody responses against sera from patients with early stage AE [19, 20].

Five distinct EgAgB isoforms (EgAgB1-5) have been synthesized from at least ten unique genes [21], among which EgAgB1 and EgAgB2 molecules have been reported to be reliable serodiagnostic biomarkers for CE [22–25]. However, conflicting data have been repeatedly observed depending on experimental conditions [22, 24, 25]. More importantly, there have been a limited number of reports regarding serological cross reactivity of AE patient sera against EgAgB molecules [15, 26]. The diagnostic reliability of EgAgBs should be re-evaluated for their actual feasibility.

In the present study, we surveyed echinococcosis patients in the Qinghai-Tibetan Plateau to better understand the epidemiological situation in this area. We diagnosed patients by abdominal US examination and collected blood samples. We subsequently analyzed differential immunoreactivity segregated to distinct EgAgB isoforms to determine their diagnostic capability.

## Methods

### Patient survey and collection of serum samples

**Fig. 1:**
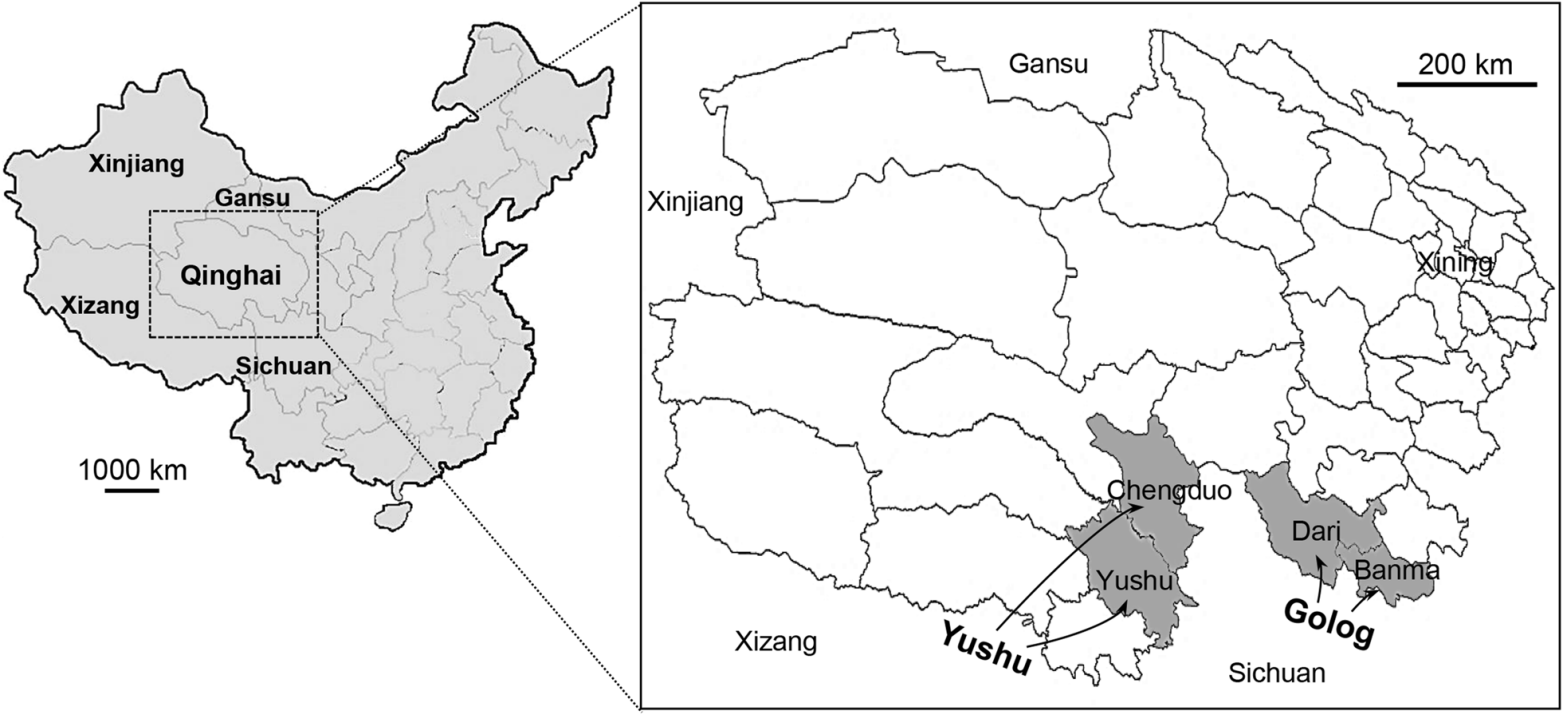
Map showing surveyed areas. We surveyed patients as part of diagnostic campaigns for echinococcoses at Chengduo and Yushu Counties (Yushu Tibetan Autonomous Prefecture) and Dari and Banma Counties (Golog Tibetan Autonomous Prefecture), Qinghai Province, China, during July and August of 2013 and 2014

During July and August of 2013 and 2014, we surveyed echinococcosis patients at Chengduo and Yushu Counties (Yushu Tibetan Autonomous Prefecture) and Dari and Banma Counties (Golog Tibetan Autonomous Prefecture), Qinghai Province, China, in a health promotion campaign. These places were highland seminomadic areas approximately 600–900 km away from Xining (Fig. 1). The study purpose was explained to local health authorities, school teachers, community leaders and residents to ensure their acceptance and participation. We screened 2856 people (male:female ratio, 1:1.12; mean age, 34.6 years; age range, 6–88 years) by abdominal US examination (Aloka SSD 500, Ganim Medical, Delaware, OH, USA). In patients with hepatic space-occupying lesion(s) compatible with echinococcoses (Additional file 1: Figure S1), power Doppler sonography was additionally conducted to observe the distribution of blood flow. Blood samples were collected from persons who understood our purpose and agreed to participate. We were able to collect blood samples from 122 patients. In addition, we collected blood samples randomly from 62 age- and sex-matched apparently healthy persons (male:female ratio, 1:1.14; mean age, 31.4 years; age range, 9–67 years) who did not show any abnormality on abdominal US (Table 1). They denied possible exposure to parasitic infections including protozoans. All patients and healthy donors enrolled in this study were dog owners from the same villages with similar occupations (semi-nomads and semi-farmers). Patients who had already been diagnosed as echinococcoses were excluded from statistical analyses [27, 28].

**Table 1:** Age and sex distribution of persons surveyed and patients identified based on abdominal ultrasonography

Age (years)	No. examined (%)			No. positive (%)			No. of healthy controls (%)		
	Male	Female	Total	Male	Female	Total	Male	Female	Total
< 10	175	182	357	4 (2.3)	4 (2.2)	8 (2.2)	1 (0.6)	2 (1.1)	3 (0.8)
10–19	358	428	786	14 (3.9)	18 (4.2)	32 (4.1)	6 (1.7)	7 (1.6)	13 (1.7)
20–29	201	245	446	7 (3.5)	6 (2.5)	13 (2.9)	3 (1.5)	3 (1.2)	6 (1.3)
30–39	289	302	591	10 (3.5)	16 (5.3)	26 (4.4)	5 (1.7)	7 (2.3)	12 (2.0)
40–49	151	161	312	13 (8.6)	15 (9.3)	28 (9.0)	7 (4.6)	7 (4.3)	14 (4.5)
50–59	95	104	199	4 (4.2)	7 (6.7)	11 (5.5)	2 (2.1)	4 (3.8)	6 (3.0)
> 60	77	88	165	10 (13.0)	6 (6.8)	16 (9.7)	5 (6.5)	3 (3.4)	8 (4.8)
Total	1346 (47.1)	1510 (52.9)	2856 (100)	62 (4.6)	72 (4.8)	134 (4.7)	29 (2.2)	33 (2.2)	62 (2.2)

### Cloning and expression of recombinant antigen B subunits (rEgAgBs)

mRNA sequences of *EgAgB*s (*B1*, EgrG_000381200; *B2*, EgrG_000381100; *B3*, EgrG_000381600; *B4*, EgrG_000381400; and *B5*, EgrG_000381800) were retrieved from GeneDB (http://www.genedb.org/Homepage/Egranulosus). Total RNA was extracted from a single ovine CE2 cyst using an RNA extraction kit (iNtRON, Seongnam, Korea). cDNA was reverse transcribed using a first strand cDNA synthesis kit (Invitrogen, Carlsbad, CA, USA). Mature domains of respective genes were amplified with gene-specific primers (Additional file 2: Table S1). PCR cycling conditions were as follows: denaturation at 95 °C for 5 min, 35 cycles of 95 °C for 1 min, 50 °C for 45 s and 72 °C for 1 min, with a final extension step at 72 °C for 5 min. Each plasmid was digested with restriction enzymes, ligated into a pGEX-6p-1 expression vector (Novagen, Cambridge, MA, USA) and transformed into *Escherichia coli* BL21 (DE3) competent cells. Recombinant proteins fused with glutathione transferase (GST) were expressed by treatment with 1 mM isopropyl β-d-1-thiogalactopyranoside (IPTG) at 37 °C for 4 h. Recombinant proteins were purified using glutathione-Sepharose 4B beads (GE Healthcare, Piscataway, NJ, USA), after which the GST-tag was removed by PreScission protease (GE Healthcare). Homogeneity of purified proteins was monitored by Tricine SDS-PAGE (10%) with Coomassie brilliant blue R-250 (CBB) staining.

### Enzyme-linked immunosorbent assay (ELISA)

After a checkerboard titration, 100 µl of each rEgAgB (1.5 µg/ml suspended in 100 mM carbonate-bicarbonate buffer, pH 9.6) was used to coat the wells of a flat-bottom 96-well microplate (Greiner Bio-One, Kremsmuenster, Austria) overnight at 4 °C. One hundred microliters of serum samples diluted 1:100 in phosphate buffered saline containing 0.05% Tween 20 (PBS/T) were incubated at 37 °C for 2 h, after which 100 µl of horseradish peroxidase-conjugated anti-human IgG antibody (1:4000 dilution in PBS/T; MP Biochemicals, Santa Ana, CA, USA) was further incubated at 37 °C for 2 h. Color reactions were developed with 100 µl of 1% *o*-phenylenediamine (Sigma-Aldrich, St. Louis, MO, USA) supplemented with 0.03% H_2_O_2_ for 20 min in the dark. Reactions were halted by adding 50 µl of 2 N H_2_SO_4_ (Sigma-Aldrich). The absorbance was measured at 450 nm on an NEO microplate reader (Biotek, Winooski, VT, USA). Sera from healthy donors and PBS/T were used as negative and blank controls, respectively. All results were determined after correction with appropriate blank. Each sample was independently assayed in triplicate.

### Statistical analyses

We used positive/negative (P/N) values to normalize data from different batches of ELISA results. Receiver operating characteristic (ROC) curves were generated by plotting sensitivity (%) *versus* 100-specificity (%). The area under the ROC curve (AUC) was employed to conduct pairwise comparisons of diagnostic performance. We determined cut-off values using ROC analyses and defined them as P/N values that rendered the highest sum of sensitivity and specificity. Graphical representations were created employing GraphPad Prism v.7.0. Accuracies of diagnostic tests such as sensitivity, specificity, Youden’s index, positive/negative likelihood ratios (PLR/NLR), and positive/negative predictive values (PPV/NPV) were determined using MedCal (free statistical calculators and diagnostic test evaluation calculator) with 95% confidence interval (CI). Student’s t-tests and one-way analysis of variance (ANOVA) were used to compare groups. Significance was set at *P* < 0.05.

## Results

### Ultrasonographic diagnosis of CE and AE patients

We surveyed echinococcosis patients at two counties each in Yushu (Yushu and Chenduo Counties) and Golog (Dari and Banma Counties), Tibetan Autonomous Prefectures (Fig. 1). A total of 2856 people were examined by abdominal US for the presence of hepatic echinococcal cysts. People aged between 10 and 40, those who were physically healthy, and with strong socioeconomic activities comprised the majority (74.8%) of the study population. Most men were half-nomadic and half-farmers while most women were housewives. The young age group less than 10 years-old constituted 12.5% of participants and those who were older than 50 years accounted for 12.7% of participants. No difference between males and females was observed in these subjects (male:female = 1:1.12; *t*_(12)_ = 0.3948, *P* = 0.6999) (Table 1).

**Fig. 2:**
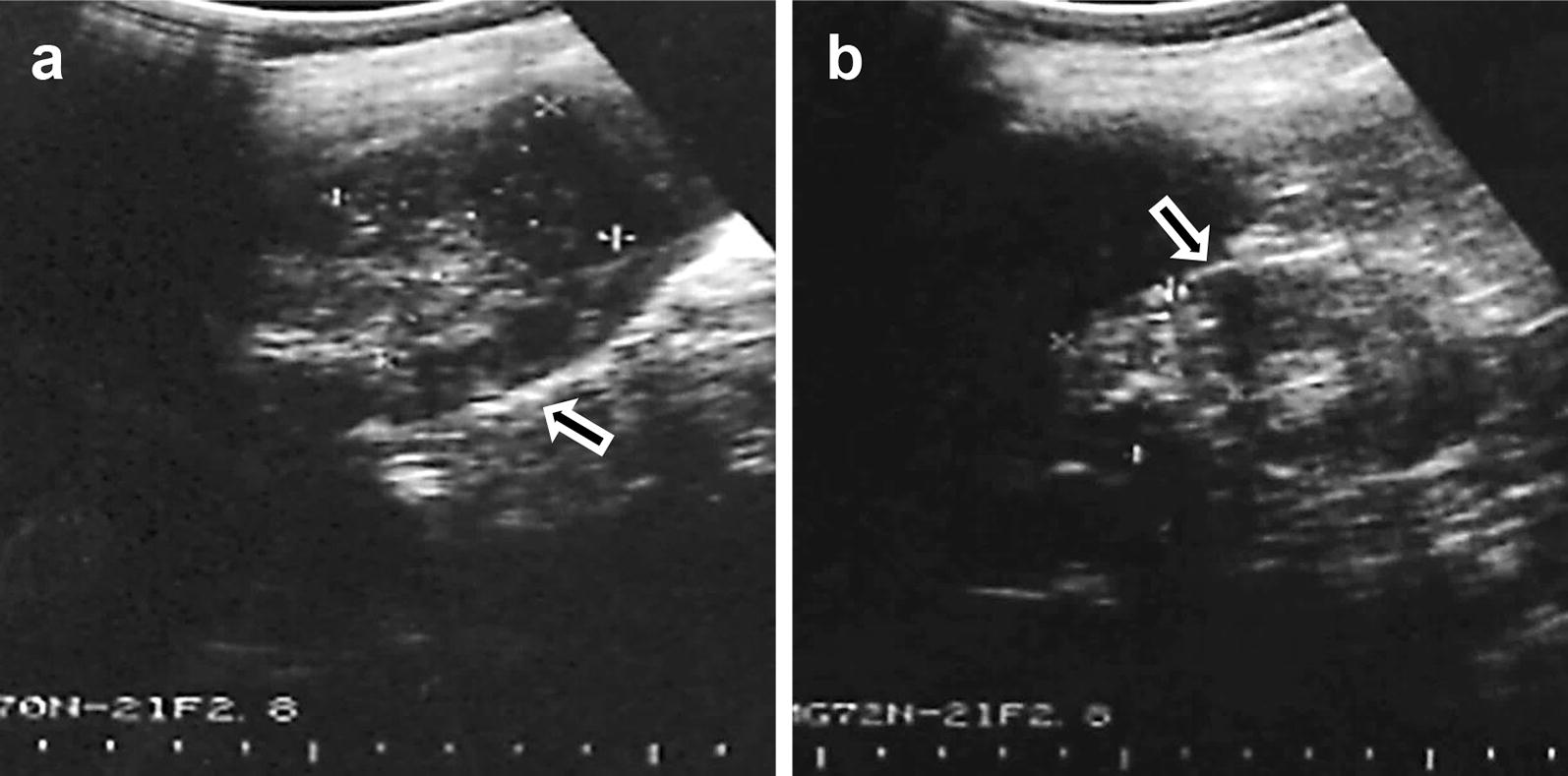
Ultrasonogram of a 48-year-old herdsman who might be concomitantly infected with cystic echinococcosis (CE) and alveolar echinococcosis (AE). **a** Ultrasonographic (US) examination revealing a 42 × 60 mm sized cystic lesion with diffuse internal calcification and hyperechoic indistinct margin (arrow) on the right hepatic lobe (CE5 stage). **b** Another view of the US scan showing a 74 × 50 mm sized cystic mass with clusters of multiple echogenic small nodules and a calcified margin (arrow) on the right hepatic lobe (early AE stage)

We were able to detect 134 new patients whose imaging scans were compatible with CE or AE, among which 115 cases showed US images probably representative of CE and 20 cases of AE [20, 29–31] (Table 2). Gender difference was not observed for disease incidence (*t*_(12)_ = 0.1622, *P* = 0.8733). A 48-year-old herdsman at Dari County (Golog Prefecture) was found to be concomitantly infected with a CE5 cyst and an early AE mass (Fig. 2), although we could not confirm his diagnosis by histopathological examination.

**Table 2:** Patients with cystic echinococcosis (CE) and alveolar echinococcosis (AE) diagnosed by ultrasonographic findings

Disease category	Stage	No. of patients (mean age, years)		
		Male	Female	Total^a^
CE	CE1	17 (33.2)	10 (20.3)	27 (28.4)
	CE2	6 (41.2)	13 (31.3)	19 (34.4)
	CE3a	5 (33.6)	5 (34.8)	10 (34.2)
	CE3b	5 (25.4)	16 (39.4)	21 (35.9)
	CE4	9 (43.6)	14 (35.9)	23 (38.7)
	CE5	10 (52.3)	5 (46.0)	15 (50.2)
Subtotal		52 (38.9)	63 (34.1)	115 (36.2)
AE	Early AE	5 (18.6)	3 (27.3)	8 (21.9)
	Advanced AE	6 (24.8)	6 (21.3)	12 (23.1)
Subtotal		11 (22.0)	9 (23.3)	20 (22.6)
Total		63 (35.9)	72 (32.7)	135^a^ (34.2)

^a^Total number exceeded 134 due to one case with double infection of both AE and CE

Representative ultrasonograms of CE patients are shown in Additional file 1: Figure S1a. Most CE1 patients revealed typical US findings of well-defined round unilocular hypodense cystic mass(es) with variable sizes (3.1–8.5 cm in diameter). Snow-flake sign and/or double-line sign were typically observed. CE2 patients exhibited multiseptated daughter cysts within a unilocular large mother cyst with size ranging between 7.8–11.1 cm in diameter. CE3a cysts showed a detachment of laminated endocyst (water-lily sign) and CE3b cysts contained solid matrix with daughter cysts (wheel-like appearance). Cyst sizes were variable (6.4–9.5 cm). CE4 patients demonstrated heterogeneous hypoechoic degenerative contents. Degenerating membranes revealed canalicular structure (ball of wool appearance). Cyst sizes varied between 6.3–8.8 cm. CE5 stage was characterized by a highly degenerative cyst with thick calcified wall. Of 115 CE patients, transitional CE3 stage was detected the most frequently (27%, 31/115 cases; 10 and 21 patients with CE3a and CE3b, respectively), followed by CE1 (*n* = 27), CE4 (*n* = 23), CE2 (*n* = 19) and CE5 stages (*n* = 15).

Active CE1 and CE2 cases were initially diagnosed at a mean age of 31.6 years (*n* = 46) while those with transitional CE3a/b stages were 34.1 years (CE3a) and 35.6 years (CE3b), respectively. Chronic inactive CE4 and CE5 stages were generally detected in patients in their late 30s to 50s (mean age of 38.7 and 50.8 years for CE4 and CE5, respectively, Table 2). Involvement of the right hepatic lobe was commonly observed (70.4%, 81/115 cases). Multiple cysts were recognized in 16 (13.9%) patients.

Among 20 cases of AE, eight patients were suspected to be in the early stage because their imaging scans disclosed a mass containing multiple echogenic small nodules (hailstorm appearance) with indistinct borders combined with or without scattered calcifications in the mass. Another 12 patients showed US findings consisting of a central hypoechogenic mass combined with peripheral hyperechoic indistinct and irregular margins that were compatible with advanced stage AE (Table 2). Sizes of these masses were variable (3.4–7.8 cm in diameter). Typical US features are presented in Additional file 1: Figure S1b. No significant difference in age at the time of diagnosis was observed between early and advanced stages (mean age: 22.3 years *versus* 23.1 years; *t*_(18)_ = 0.8285, *P* = 0.4183). All patients had a single lesion predominantly in the right lobe (80%, 16/20 cases).

Of 134 echinococcosis patients, 122 patients (110 CE and 12 AE) agreed to provide blood samples (Table 3). These samples were used to evaluate diagnostic performance of diverse rEgAgB isoforms.

**Table 3:** Serodiagnostic performance of recombinant *E. granulosus* antigen Bs (rEgAgBs) against serum samples from different stages of cystic echinococcosis (CE) and alveolar echinococcosis (AE) cases

	No. tested	No. positive (%)				
		rEgAgB1	rEgAgB2	rEgAgB3	rEgAgB4	rEgAgB5
CE1	26	21 (80.8)	24 (92.3)	23 (88.5)	16 (61.5)	21 (80.8)
CE2	19	16 (84.2)	18 (94.7)	18 (94.7)	12 (63.2)	14 (73.7)
CE3a	10	9 (90.0)	10 (100)	10 (100)	9 (90.0)	9 (90.0)
CE3b	20	20 (100)	20 (100)	20 (100)	16 (80.0)	15 (75.0)
CE4	22	18 (81.8)	20 (90.9)	18 (81.8)	15 (68.2)	20 (90.9)
CE5	13	9 (69.2)	10 (76.9)	10 (76.9)	7 (53.8)	9 (69.2)
AE	12	6 (50.0)	4 (33.3)	4 (33.3)	9 (75.0)	10 (83.3)
Controls	62	0 (0)	0 (0)	0 (0)	9 (14.5)	17 (27.4)
Sensitivity	110	93 (84.5)	102 (92.7)	99 (90.0)	75 (68.2)	88 (80.0)
Specificity	74	6 (91.9)	4 (94.6)	4 (94.6)	18 (75.7)	27 (63.5)
AUC ± SE		0.9216 ± 0.02007	0.9613 ± 0.01283	0.9517 ± 0.01641	0.8049 ± 0.03247	0.7693 ± 0.03562
95% CI		0.8823–0.9610	0.9362–0.9864	0.9196–0.9839	0.7413–0.8686	0.6995–0.8391
Cut-offs^a^		0.1625	0.1760	0.2000	0.1705	0.1190
PLR		10.43	17.15	16.65	2.8	2.19
NLR		0.17	0.08	0.11	0.42	0.31
PPV (%)		93.9	96.2	96.1	80.7	76.5
NPV (%)		80.0	89.7	86.4	61.5	68.1
Youden’s index		0.76	0.87	0.85	0.44	0.44

^a^Cut-off values were determined by analysis of ROC curves

*Abbreviations*: AE, alveolar echinococcosis; AUC, area under the ROC curve; CE, cystic echinococcosis; CI, confidence interval; NLR, negative likelihood ratio; NPV, negative predictive value; rEgAgB, recombinant *E. granulosus* antigen B; SE, standard error; PLR, positive likelihood ratio; PPV, positive predictive value

### Immunoreactivity profiles of rEgAgBs against patients’ sera at different CE stages

We retrieved representative sequences encoding EgAgB1-5, whose sequence identities ranged between 37.65–71.91% at the mRNA level (Additional file 3: Figure S2a). We expressed recombinant proteins in a prokaryotic system after fusion with GST. The GST-tag was cleaved with human rhinovirus type 14 3C protease. Tricine SDS-PAGE (10%) revealed that rEgAgB1 to rEgAgB5 migrated along with their predictive molecular weights of 7.6, 8.2, 7.9, 8.2 and 7.5 kDa, respectively. Their purity exceeded 98.5% by densitometric analyses (Additional file 3: Figure S2b).

**Fig. 3:**
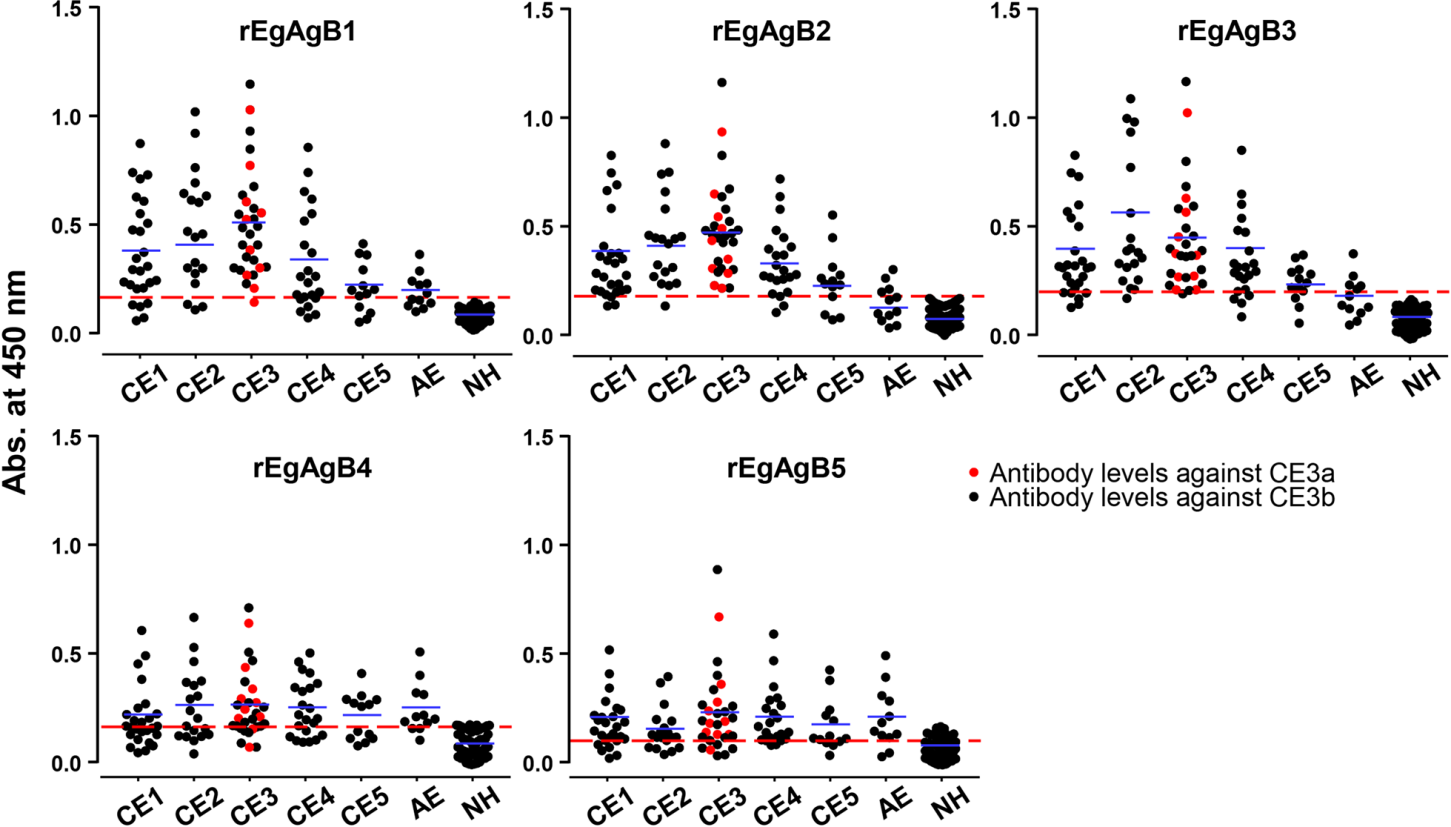
Specific IgG antibody levels in sera of patients with different stages of cystic echinococcosis (CE) and alveolar echinococcosis (AE) and those from healthy controls (NH) against rEgAgB1-5. The Y-axis displays absorbance values of detected sera. The X-axis indicates the following sera groups: CE1 (*n* = 26); CE2 (*n* = 19); CE3 (*n* = 30; 10 and 20 samples of CE3a and CE3b, respectively); CE4 (*n* = 22); CE5 (*n* = 13); AE (*n* = 12); and NH (*n* = 62). The cut-off values for respective rEgAgBs are determined by receiver operating characteristic (ROC) analysis (red dotted lines). Horizontal blue bars denote average specific antibody levels in each group

**Fig. 4:**
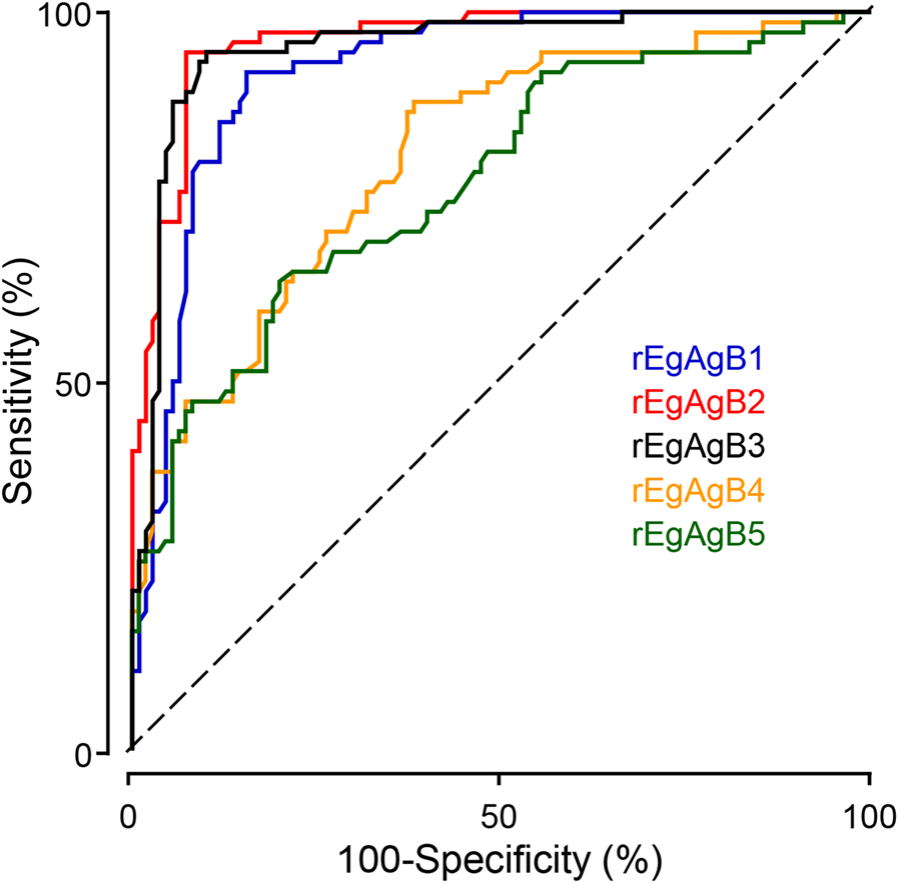
Receiver operating characteristic (ROC) curves for rEgAgB1-5 assay in serum samples by ELISA. These curves represent plots of sensitivity (%) *versus* 100-specificity (%) for respective rEgAgBs. The reference line is also shown (dotted diagonal line). ROC curves are used to determine cutoff values and areas under the curve (AUC) each for rEgAgB isoforms against serum samples from patients with CE (*n* = 110)

We evaluated differential immunoreactivity of the distinct rEgAgB isoforms employing sera from patients with different CE stages. rEgAgB1, rEgAgB2 and rEgAgB3 showed fairly high antibody reactivity whereas rEgAgB4 and rEgAgB5 exhibited relatively weak antibody responses (Fig. 3). The optimal cut-off values determined by analyzing the ROC curves demonstrated that absorbance of 0.1625 for rEgAgB1, 0.1760 for rEgAgB2 and 0.2000 for rEgAgB3 distinguished the positive and negative reactions with 95% CIs of 0.8823–0.9610 (rEgAgB1), 0.9362–0.9864 (rEgAgB2) and 0.9196–0.9839 (rEgAgB3). These ROC curves further indicated that rEgAgB2 was the most promising biomarker, followed by rEgAgB3 and rEgAgB1 (Fig. 4). Youden’s index was 0.87 for rEgAgB2, 0.85 for rEgAgB3 and 0.76 for rEgAgB1. Overall diagnostic sensitivity and specificity of rEgAgB1-3 ranged between 84.5–92.7% and 91.9–94.6%, respectively. PLR/NLR values of rEgAgB1, 2 and 3 were 10.43/0.17, 17.15/0.08 and 16.65/011, respectively. Relevant statistical variables such as sensitivity/specificity, AUC, 95% CI, Youden’s index, PLR/NLR and PPV/NPV of individual rEgAgBs are summarized in Table 3.

Specific antibody levels between CE3a (*n* = 10) and CE3b (*n* = 21) for rEgAgBs were not significantly different (rEgAgB1: *t*_(29)_ = 0.3441, *P* = 0.7333; rEgAgB2: *t*_(29)_ = 0.4481, *P* = 0.6574; rEgAgB3: *t*_(29)_ = 0.0143, *P* = 0.9887; rEgAgB4: *t*_(29)_ = 0.7724, *P* = 0.4461; rEgAgB5: *t*_(29)_ = 0.2995, *P* = 0.7667) (Fig. 3).

A previous study has been shown that EgAgB1 might be a promising biomarker for determining cyst viability [16]. However, when we compared the differential diagnostic performance of rEgAgB1 to discriminate active infections from chronic stages, rEgAgB1 did not show such a significant difference, although rEgAgB1 demonstrated lower antibody responses against sera from chronic stages of CE4 (*F*_(3,65)_ = 2.4609, *P* = 0.0935) and CE5 (*F*_(3,38)_ = 2.2949, *P* = 0.7464) to some extent (Fig. 3).

We further assessed cross-reactivity attributed to different rEgAgB proteins to analyze serodiagnostic feasibility. As shown in Table 3 and Fig. 3, 50% of serum samples from patients with AE exhibited false positive reactions against rEgAgB1. Conversely, 33.3% of sera from AE cases showed cross-reactivity with rEgAgB2 or rEgAgB3. rEgAgB4 and rEgAgB5 displayed 75 and 83.3% cross-reactions against serum samples from AE patients examined. Serum samples obtained from age- and sex-matched healthy donors who were the same villagers with similar occupations and dog owners showed no cross-reaction against rEgAgB1, rEgAgB2 or rEgAgB3. Conversely, these sera demonstrated cross-reactions against rEgAgB4 and rEgAgB5 at rates of 14.5% (9/62 samples) and 27.4% (17/62 sera), respectively.

## Discussion

In this study, we surveyed the endemicity of echinococcoses by abdominal US examination at remote mountainous seminomadic area of southeastern Qinghai-Tibetan Plateau (Golog and Yushu Tibetan Autonomous Prefectures). We also collected serum samples to analyze specific antibody levels against diverse rEgAgBs to evaluate their diagnostic feasibility when both CE and AE are co-endemic. The overall infection rate of echinococcoses reached up to 4.7% (115 CE cysts and 20 AE masses in 134 patients) among the 2856 people examined. The majority of patients suffered from CE (4.0%) and those suffering from AE constituted a minor fraction (0.7%). When we assessed the diagnostic applicability of rEgAgB isoforms employing serum samples from these patients, rEgAgB2 demonstrated the most promising potential as a CE biomarker. rEgAgB3 and rEgAgB1 also exhibited consistent diagnostic performance. Conversely, rEgAgB4 and rEgAgB5 showed low sensitivity with high cross reactions against serum samples from AE patients as well as those from healthy donors. We could not analyze immune responses of one patient who might be concomitantly infected with CE and AE due to his refusal to draw a blood sample.

Both CE and AE are known to be endemic in Qinghai Province and are associated with significant disability-adjusted life years. However, reports on the actual prevalence rate of echinococcoses in the Qinghai-Tibetan Plateau, based on US examination, has been relatively limited [27, 28, 32]. Our survey strongly suggests that public health concerns related to echinococcoses in this area might be more serious than hitherto considered [8, 27, 28, 32]. The prevalence of echinococcoses has recently been reported, ranging between 1.7–2.1% (0.6–0.8% for CE and 1.1–1.3% for AE) among school children in the Qinghai-Tibetan area [27, 28]. We extended our survey to adult populations to figure out general prevalence of echinococcoses in this endemic area. Our results demonstrated somewhat different epidemiological characteristics compared to previous studies [27, 28]. We observed a higher prevalence rate of echinococcoses (4.7%) than those in earlier studies. We surmised that our study may not have detected all cases because we did not conduct lung examinations, which is the second most predilection site for CE [1]. There was no gender difference in our study, while previous studies have shown a high prevalence in female children [27, 28]. Although the number of AE patients detected was not statistically significant, patients with AE might suffer from the disease starting at earlier ages (age range: 8–48 years; mean: 22.7 years) compared to those with CE (age range: 6–88 years; mean: 37.5 years) (Tables 1, 2). CE patients were largely (67.5%) diagnosed at active and transitional stages of CE1-3, while a considerable proportion (60%) of AE patients were detected at an advanced stage (Table 2).

We observed fewer AE cases compared to those in previous studies [27, 28, 32]. This result might be attributable to the fact that we examined reasonably small numbers of school children comparted to previous studies. Moreover, patients who had already started disease treatment were excluded from statistical analyses. Alternatively, the endemicity of AE might be restricted to relatively small area, mostly confined to Golog Prefecture (Dari and Banma Counties) as reported previously [27, 28, 32].

Golog and Yushu Prefectures are seminomadic areas where lots of ungulates graze in a wide meadow. Dogs are raised to manage cattle. In the nomadic way of life, women are responsible for several households during which they might be exposed to dogs and their feces contaminated with CE eggs. They might become infected with CE. Men might be exposed to *E. granulosus* eggs while grazing cattle with their shepherd dogs in the steppe [32, 33]. These environmental, ecological and human geographical characteristics might have contributed to the active transmission of CE in these areas.

It is interesting to note that the transmission mode of AE in the Qinghai-Tibetan Plateau seems to be different from that in European countries. In European communities, the adult population might be more likely to contract AE while gardening with bare hands, hunting in the forest, or picking mushrooms in grasslands associated with fox habitats [34–36]. Conversely, AE had a high infection rate in the young age group in the Qinghai-Tibetan Plateau (Table 2). This epidemiological situation corroborated well with previous studies [27, 28]. Most children might be infected with AE by dirt-eating habits during playing in the steppe and/or helping parents in daily activities such as taking care of livestock and household chores. They usually gather dry animal excrement, which might be mixed with that of dogs, as major sources of fuel materials. These traditional social behaviors might have played major roles in the transmission of AE among children in these areas [37, 38].

Our previous analysis involving the immunoproteome profile of native EgAgB proteins has demonstrated that EgAgB1 comprises the most abundant fraction in both CE1 and CE2 HF and that EgAgB1 is the most sensitive antigen [15]. Neither EgAgB2 nor EgAgB3 exhibited such sensitive responses but showed positive reactions mainly to sera from patients with CE2 and CE3 stages, during which host immune responses might be highly active [15]. Other serodiagnostic studies using synthetic EgAgBs have also indicated the high antibody capturing activity of EgAgB1 [16, 25]. However, when we analyzed differential antibody reactivity of each recombinant protein, the order of diagnostic performance from high to low was: rEgAgB2 > rEgAgB3 > rEgAgB1 > rEgAgB4 > rEgAgB5 (Figs. 3, 4). Furthermore, rEgAgB2 and rEgAgB3 exhibited less cross-reaction against sera from patients with AE than rEgAgB1 (Table 3). Previous studies with rEgAgB2 have also revealed a reliable diagnostic applicability [14, 22, 23]. Moreover, a multimeric form of EgAgB2 protein (B2t) manipulated by tandem ligation of a single nucleotide sequence of mature EgAgB2 domain has shown to significantly enhance diagnostic feasibility [14]. Although the antigenic reactivity of native EgAgB2 was not satisfactory probably due to its low expression levels in native HF [15, 39], the EgAgB2 molecule might have epitope(s) specific to CE. The generation of a chimeric protein through the combination of domain(s) harboring specific epitopes might have an advantage not only for improving CE serodiagnosis, but also for differential diagnosis between CE and AE. The use of relevant chimeric antigen significantly improved the serodiagnostic reliability [40].

Synthetic EgAgB1 has been reported to be a promising biomarker for determining cyst viability, thus providing a clue to differentially diagnose active stage CE from chronic cases [16]. However, rEgAgB1 did not show such a significant difference, although rEgAgB1 demonstrated lower antibody responses against sera from chronic stages of CE4 and CE5 (Fig. 3). This conflicting observation suggests that antibody reactivity of rEgAgB1 might be somewhat different depending on the serum sample tested. Antibody responses mounted to a certain antigen(s) might reflect several important factors, including immunological status of the infected individual and biological reactivity of parasite molecules during host-parasite interactions. We assumed that serological responsiveness might be more importantly determined by host factors than by parasite factors [41]. To precisely address the differential antibody reactivity mounted to each EgAgB isoform, host factors involved in serological responsiveness should be carefully considered in future investigations.

## Conclusions

Our results indicate that public health concerns related to echinococcoses in the Qinghai-Tibetan area seem to be more serious than previously considered. Abdominal US should still be the diagnosis of choice. Detection of specific antibody levels against rEgAgB2, rEgAgB3 and/or rEgAgB1 might provide supportive data for the diagnosis of CE. However, in areas where both CE and AE are co-endemic, serological tests should be carefully interpreted for differential diagnosis. The transmission mode of AE in the Qinghai-Tibetan Plateau might be different from that in other communities. Further studies are urgently needed to assess transmission potentials of echinococcoses in this area. Interventions for disease control are also warranted to ensure patients’ health and reduce social burdens.

## Additional files

**Additional file 1: Figure S1.** Representative ultrasonographic findings of cystic echinococcosis (CE) and alveolar echinococcosis (AE) patients in this study. **a** Imaging scans of CE patients. CE1 stage shows well-defined cyst wall with floating and sedimenting echoes (snow-flake sign). Ultrasonograms of some patients also show double-line sign. CE2 stage demonstrates multiple daughter cysts within a unilocular large cyst. CE3a stage displays detachment of the pericystium and CE3b exhibits multiloculated daughter cysts with solid matrix. CE4 typically shows canalicular structure (ball of wool appearance). CE5 stage reveals highly degenerative cyst with calcified wall. **b** Ultrasonographic findings of AE cases. Sonogram of early AE case demonstrates clusters of multiple echogenic small nodules (hailstorm patterns) with indistinct margin with/without punctate calcifications. Advanced cases reveal a large central hypoechogenicity combined with peripheral hyperechoic indistinct and irregular border.

**Additional file 2: Table S1.** Gene-specific oligonucleotide primers used.

**Additional file 3: Figure S2.** **a** Comparison of homology values among EgAgB1-5 at mRNA level. **b** Expression and purification of recombinant EgAgBs (rEgAgBs). rEgAgBs fused with GST were induced with 1 mM IPTG and purified using GSH-Sepharose 4B. GST-tags were removed by PreScission protease. The homogeneity of purified proteins was analyzed by Tricine SDS-PAGE (10%) with CBB staining. Lanes 1–5 represent respective recombinant proteins. *M*_r_, molecular weight in kDa.

## Data Availability

The data supporting the conclusions are included within the article and its additional files. The raw data used or analyzed during the study are available from the corresponding author upon reasonable request.

## Abbreviations

AE: alveolar echinococcosis
AUC: area under the ROC curve
CE: cystic echinococcosis
CI: confidence interval
EgAgB: *E. granulosus* antigen B
EgAg5: *E. granulosus* antigen 5
ELISA: enzyme-linked immunosorbent assay
EmAgB: *E. multilocularis* antigen B
HF: hydatid fluid
NLR: negative likelihood ratio
NPV: negative predictive value
PBS/T: phosphate buffered saline containing 0.05% Tween 20
PLR: positive likelihood ratio
PPV: positive predictive value
rEgAgB: recombinant EgAgB
ROC: receiver operating characteristic
US: ultrasonography
