# Supplementary figures and images for: Survey of echinococcoses in southeastern Qinghai Province, China, and serodiagnostic insights of recombinant Echinococcus granulosus antigen B isoforms
Source: Parasit Vectors. 2019 Jun 26;12:323. doi: 10.1186/s13071-019-3569-6 (PMC6593596; doi:10.1186/s13071-019-3569-6)

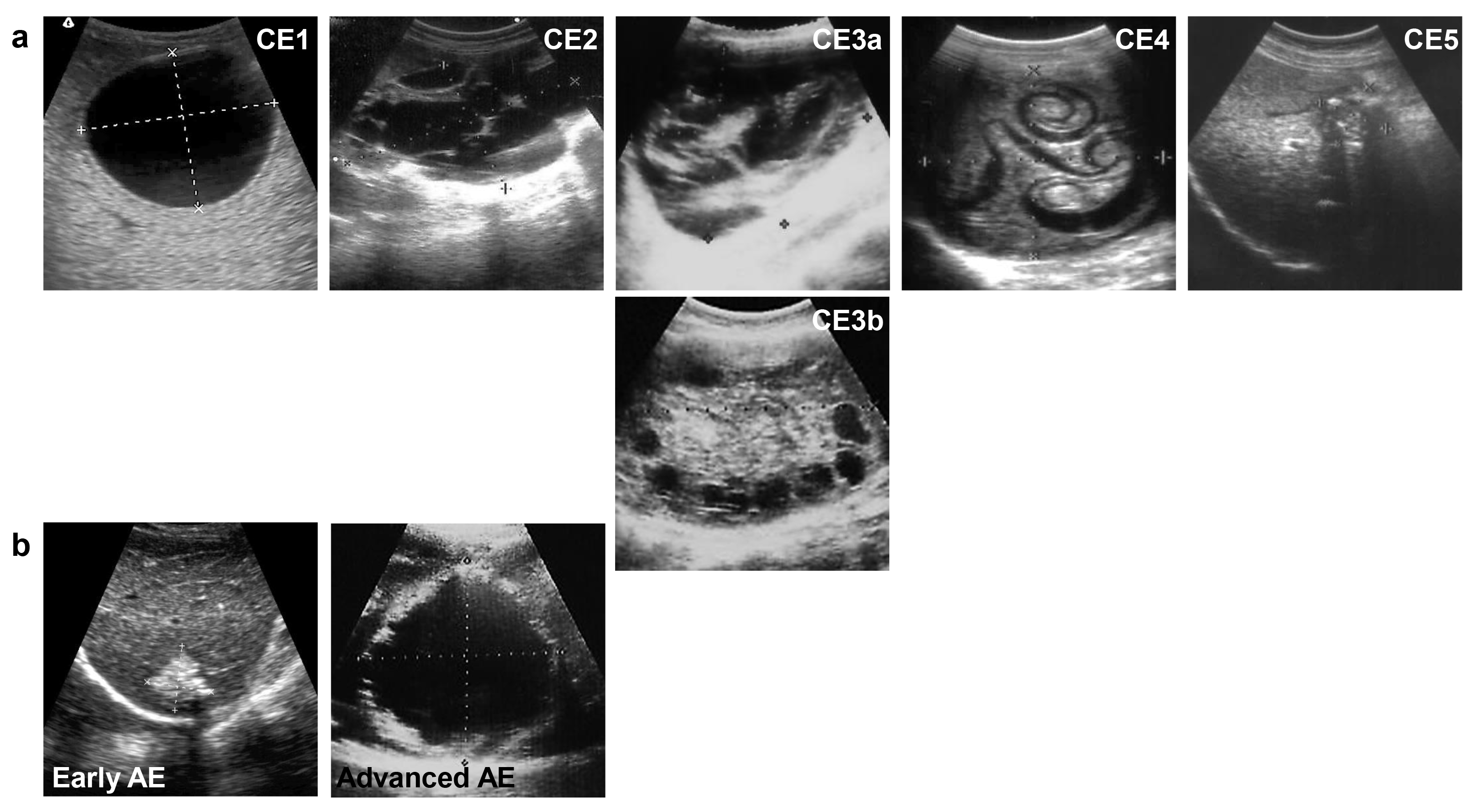

Supplement: Supplementary file 1 — Additional file 1: Figure S1. Representative ultrasonographic findings of cystic echinococcosis (CE) and alveolar echinococcosis (AE) patients in this study. a Imaging scans of CE patients. CE1 stage shows well-defined cyst wall with floating and sedimenting echoes (snow-flake sign). Ultrasonograms of some patients also show double-line sign. CE2 stage demonstrates multiple daughter cysts within a unilocular large cyst. CE3a stage displays detachment of the pericystium and CE3b exhibits multiloculated daughter cysts with solid matrix. CE4 typically shows canalicular structure (ball of wool appearance). CE5 stage reveals highly degenerative cyst with calcified wall. b Ultrasonographic findings of AE cases. Sonogram of early AE case demonstrates clusters of multiple echogenic small nodules (hailstorm patterns) with indistinct margin with/without punctate calcifications. Advanced cases reveal a large central hypoechogenicity combined with peripheral hyperechoic indistinct and irregular border. [file 13071_2019_3569_MOESM1_ESM.tif]

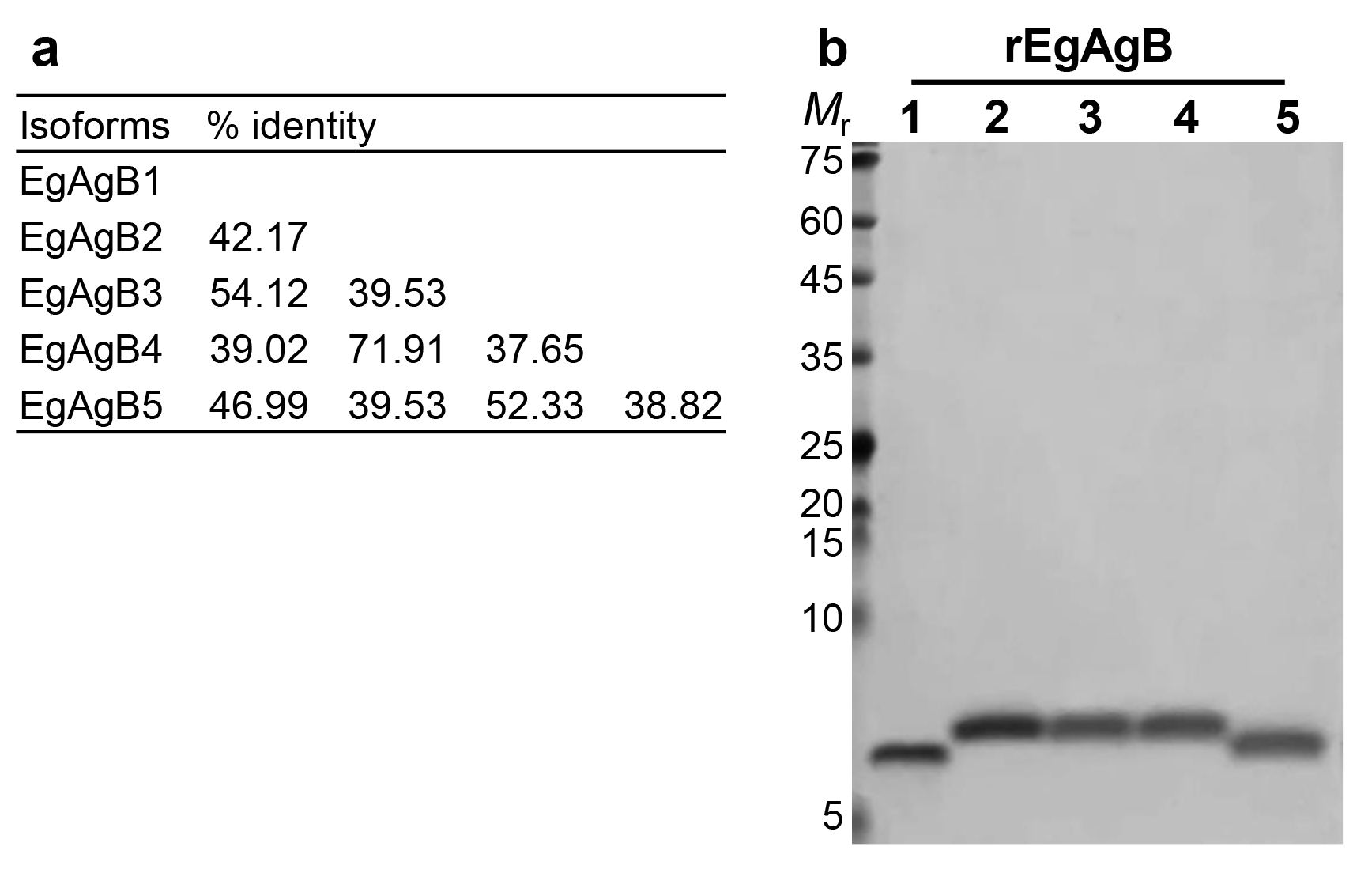

Supplement: Supplementary file 3 — Additional file 3: Figure S2. a Comparison of homology values among EgAgB1-5 at mRNA level. b Expression and purification of recombinant EgAgBs (rEgAgBs). rEgAgBs fused with GST were induced with 1 mM IPTG and purified using GSH-Sepharose 4B. GST-tags were removed by PreScission protease. The homogeneity of purified proteins was analyzed by Tricine SDS-PAGE (10%) with CBB staining. Lanes 1–5 represent respective recombinant proteins. Mr, molecular weight in kDa. [file 13071_2019_3569_MOESM3_ESM.tif]
