# Supplementary material for: Survey of echinococcoses in southeastern Qinghai Province, China, and serodiagnostic insights of recombinant Echinococcus granulosus antigen B isoforms
Source: Parasit Vectors. 2019 Jun 26;12:323. doi: 10.1186/s13071-019-3569-6 (PMC6593596; doi:10.1186/s13071-019-3569-6)
Supplement: Supplementary file 2 — Additional file 2: Table S1. Gene-specific oligonucleotide primers used. [file 13071_2019_3569_MOESM2_ESM.docx]

**Additional file 2: Table S1** Gene-specific oligonucleotide primers used.

| **Gene name (EgrG_)** | **Direction** | **Oligonucleotide (5'→3')*** | **Restriction enzyme** |
| --- | --- | --- | --- |
| *EgAgB1* | Forward | GCGGATCCGATGATGGCCTCACCTCG | *Bam*HI |
| (000381200) | Reverse | CGCTCGAGCTATTCACCTTCAGCAAT | *Xho*I |
| *EgAgB2* | Forward | GCGGATCCAAAGATGAGCCAAAAGCA | *Bam*HI |
| (000381100) | Reverse | CGCTCGAGTTACTTTGAATCATCATC | *Xho*I |
| *EgAgB3* | Forward | GCGGATCCGATGATGATGATGATGAA | *Bam*HI |
| (000381600) | Reverse | CGCTCGAGCTACTCATCCTCTTTAAC | *Xho*I |
| *EgAgB4* | Forward | GCGGATCCAAAGCTGAACCCGAGAGA | *Bam*HI |
| (000381400) | Reverse | CGCTCGAGTTACTTTAAATCATCCTC | *Xho*I |
| *EgAgB5* | Forward | GCGGATCCGAAGATGACATCGATTCG | *Bam*HI |
| (000381800) | Reverse | CGCTCGAGCTACTCCCCTTCATCCAT | *Xho*I |

*Restriction sites are underlined.
